# Fire regime on a cultural landscape: Navajo Nation

**DOI:** 10.1002/ece3.4470

**Published:** 2018-09-12

**Authors:** Lionel Whitehair, Peter Z. Fulé, Andrew Sánchez Meador, Alicia Azpeleta Tarancón, Yeon‐Su Kim

**Affiliations:** ^1^ School of Forestry Northern Arizona University Flagstaff Arizona

**Keywords:** climate, Diné, drought, fire history, Indigenous, tribal lands, wildfires

## Abstract

Fire has played an important role in the evolutionary environment of global ecosystems, and Indigenous peoples have long managed natural resources in these fire‐prone environments. We worked with the Navajo Nation Forestry Department to evaluate the historical role of fire on a 50 km^2^ landscape bisected by a natural mountain pass. We used fifty 5‐ha circular plots to collect proxy fire history data on fire‐scarred trees, stumps, logs, and snags in a coniferous forest centered on a key mountain pass. The fire history data were categorized into three groups: All (all 50 plots), Corridor (25 plots closest to Buffalo Pass drainage), and Outer (remaining 25 plots, farther from pass). We assessed spatial and temporal patterns of fire recurrence and fire‐climate relationships. The landscape experienced frequent fires from 1644, the earliest fire date with sufficient sample depth, to 1920, after which fire occurrence was interrupted. The mean fire interval (MFI) for fire dates scarring 10% or more of the samples was 6.25 years; there were 13 large‐scale fires identified with the 25% filter with an MFI of 22.6 years. Fire regimes varied over the landscape, with an early reduction in fire occurrence after 1829, likely associated with pastoralism, in the outer uplands away from the pass. In contrast, the pass corridor had continuing fire occurrence until the early 20th century.*Synthesis*. Fires were synchronized with large‐scale top‐down climatic oscillations (drought and La Niña), but the spatially explicit landscape sampling design allowed us to detect bottom‐up factors of topography, livestock grazing, and human movement patterns that interacted in complex ways to influence the fire regime at fine scales. Since the early 20th century, however, fires have been completely excluded. Fuel accumulation in the absence of fire and warming climate present challenges for future management.

## 1 INTRODUCTION

Fire has played an important role in the evolutionary environment of global ecosystems, and Indigenous peoples have long managed natural resources in these fire‐prone environments (Davidson‐Hunt, 2003; Ens et al., 2015; Marsden‐Smedley & Kirkpatrick, 2000; Mistry et al., 2005). The semi‐arid forests of southwestern North America, with a long history of Indigenous occupation in fire‐prone landscapes, provide an excellent opportunity in which to assess the role of fire in complex interactions of people and their environments (Fulé, Ramos‐Gómez, Cortés‐Montaño, & Miller, 2011). After centuries of pressure for changes in land uses and management practices from the Euro‐American majority in the U.S.A. and Mexico, only a few Indigenous societies in this region have maintained or restored the role of frequent surface fire regimes in dry coniferous forests (Fulé et al., 2011; Pyne, 2014; Stan, Fulé, Ireland, & Sanderlin, 2014; Weaver, 1951). As a result of extended fire exclusion, changes in land use and management practices, as well as warming climate, uncharacteristically severe wildfires are increasingly affecting southwestern landscapes and Native nations are bearing substantial damage to natural resources and traditional ways of life. Several large fires in the southwestern region occurred on Native nations, such as the Rodeo‐Chediski (2002) and Wallow (2011) fires on White Mountain Apache lands, the 2012 Las Conchas fire at Santa Clara Pueblo, and the 2014 Asaayi Lake fire on the Navajo Nation.

Changes in fire regimes have been widely studied in western North America, with the greatest number of study sites in the southwestern United States (Falk et al., 2011). However, only 3% percent of fire‐scarred tree chronologies in Arizona and New Mexico represent tribal forests (our calculation from studies reported in Falk et al., 2011), although tribal forests and woodlands are about 4.7 million ha, which is half of the size of the area that the US Forest Service manages in the southwest (Mason, 2014). The lack of attention to fire regimes on Native American lands is of particular concern because Native citizens are often closely reliant on natural resources and thus relatively vulnerable to catastrophic change (Voggesser, Lynn, Daigle, Lake, & Rance, 2013). Also, understanding changes in Indigenous management practices and their impacts can help us better manage resilient forests in fire‐prone environments under changing climates, as suggested by application of traditional fire knowledge for ecological restoration at a variety of sites around the world (Huffman, 2013). Among the few studies that have been carried out on tribal lands, fire regimes were found to differ from the general trends in the Southwest (Savage & Swetnam, 1990; Stan et al., 2014), highlighting the value of increased collaboration with Native nations for further research.

Past fire regimes in ponderosa pine and mixed‐conifer forests of the southwest were characterized by frequent, relatively low‐severity fires with mean fire intervals (MFI) ranging from 2 to 20 years (see regional summary in Swetnam & Brown, 2011). These fire events were often synchronized across multiple mountain regions by climatic oscillations, particularly the El Niño‐Southern Oscillation (ENSO; Grissino‐Mayer, Romme, Lisa Floyd, & Hanna, 2004; Swetnam & Betancourt, 1990). The arrival of Euro‐American colonists coupled with livestock grazing, logging, and fire suppression policies promoted a decline in fires beginning in the late 19th century (Ryan, Knapp, & Varner, 2013; Savage & Swetnam, 1990). Since the exclusion of fire from the landscape, there has been an accumulation of forest fuels (Hurteau, Bradford, Fulé, Taylor, & Martin, 2014). Combined with heavy contiguous fuel loads and warming climate, this has resulted in larger and more severe fires (Dillon et al., 2011; Westerling, Hidalgo, Cayan, & Swetnam, 2006).

The Navajo (Diné) Nation of the Southwest stands out due to a relatively early interruption of the fire regime circa 1830 in the Chuska Mountains, believed to be due to the increase in sheep grazing by Navajo people (Savage & Swetnam, 1990). In the same region, Guiterman (2016) confirmed a post‐1830 fire decline in study sites where grazing was likely, based on water availability. Other sites, which likely had less use, did not show a decline in fire occurrence until circa 1870, when almost all fires ceased, similar to most other southwestern forests (Guiterman, 2016).

We carried out a systematic grid‐based, spatially explicit sampling of fire‐scarred trees on a large landscape (50 km^2^) surrounding a topographic feature, Buffalo Pass, in the previously unstudied Lukachukai Mountains, which comprise the northern portion of the Chuska Mountains. Buffalo Pass is a natural corridor connecting Arizona to New Mexico. Historically and today, it serves as a passageway in crossing the mountain range. Our objectives for the study were to determine the fire regime for the Lukachukai Mountains landscape in order to test the following hypotheses: (H_1_) Fires declined in our study site in the 1830s due to pastoralism, as suggested by other studies in the Chuska Mountains, (H_2_) Buffalo Pass corridor had a higher frequency of fire occurrence than the surrounding outer uplands, due to topographic influences of the pass itself as well as high human use which is often associated with high rates of ignition (Allen, 2002). Finally, we hypothesized that (H_3_) forest fires within the Lukachukai Mountains were synchronized with climatic variation such as drought and the El Niño/Southern Oscillation, as commonly observed in the Southwest (Swetnam & Betancourt, 1990).

## 2 MATERIALS AND METHODS

### 2.1 Study area

The study area comprised the high terrain of the northern portion of the Chuska Mountains, often called the Lukachukai Mountains, located on the Navajo Nation. The Chuska Mountain range is situated within the Colorado Plateau, encompassing the northeastern region of Arizona and extending into northwestern New Mexico. Soils are primarily sandy loam soils derived from Chuska Sandstone parent material. Dispersed throughout the mountain range are pyroclastic lava flows and protrusions from the Pliocene Navajo‐Hopi volcanic region (Appledorn & Wright, 1957). The total extent for our Lukachukai study site is ~50 km^2^, with an elevational range of approximately 2,340–3,710 m. *Pinus ponderosa* (Lawson & C. Lawson) is the dominant tree species, along with patches of *Populus tremuloides* (Michx.), *Quercus gambelii* (Nutt.), and mixed conifer stands including *Pseudotsuga menziesii* var. *glauca* (Beissn.) and *Abies concolor* (Gord. & Glend.) *Lindl*. ex *Hildebr* on the north‐facing slopes. The study area is centered on Buffalo Pass, a topographical watershed divide, and travel corridor between Arizona and New Mexico. Average annual precipitation in the community of Lukachukai (elevation: 1,996 m, approximately 8.8 km southwest of study site) is 240 mm (1914–2010) with average winter snowfall of 43 cm (Western Regional Climate Center, 2010). Precipitation follows a bimodal annual pattern with winter precipitation influenced by the El Niño‐Southern Oscillation (ENSO) and summer precipitation in the form of monsoonal rain from July to September. Historic fire regimes in the region are linked to La Niña, the negative phase of ENSO (Swetnam & Betancourt, 1990).

Management includes sheep grazing beginning in the early 19th century and continuing to the present day on an annual cycle from June to September for the high country of the Chuska Mountain range (Savage & Swetnam, 1990; Strawn & Littrell, 2007). There are large open meadows dispersed throughout the study site with a small perennial stream running through the Buffalo Pass drainage. Water tanks, livestock herds, ranchers, sheepherders, and ancestral homesteads are found throughout the study site, evidence of grazing, and social influences that are still occurring on the landscape. Timber harvesting on the Navajo Nation began in the 1880s (Einbender‐Velez, 1993). The Navajo Forest Products Industries reached the level of harvesting and processing 40–50 million board feet of ponderosa yearly (10‐year Forest Management Plan, 1983) before its closure in the early 1990s; remnant stumps from these harvests are present throughout the site. According to the management plan, fire management for this region of the Navajo Nation is based on a policy of fire suppression, although limited prescribed burning is carried out on a few areas.

### 2.2 Field sampling

We selected grid‐based sampling in order to systematically assess temporal and spatial patterns over a large contiguous landscape (Farris, Baisan, Falk, Yool, & Swetnam, 2010; Farris et al., 2013; Fulé, Moore, & Covington, 1997; O′Connor, Falk, Lynch, & Swetnam, 2014). We created a 1 × 1 km grid of fifty plots within the study area starting from a randomly selected point. At each gridpoint, we created a circular 5‐ha plot (126.2 m radius) within which we searched for fire‐scarred trees. We aimed to collect approximately 4–6 samples per plot. When more scarred trees were available, we selected those which had the highest number of scars to best capture the full sequence of past fire events. Tree structures known to record the oldest fires, such as fallen logs, were sampled when available (Yocom Kent & Fulé, 2015). Past harvesting had removed the majority of old trees, so most samples were taken from stumps. Fire scars were almost never encountered on living trees, but we did sample a partial section from two living trees; this technique causes minimal injury (Heyerdahl & McKay, 2017). The landscape was dominated by coniferous forest but in some cases, the plot centers fell in meadows, rocky areas, or wetlands. Our protocol for fire‐scar collection was that such plots were moved 150 m north of grid center and continued in each cardinal direction if no samples were obtained. In total, the area searched for fire‐scarred trees was 50 × 5 ha = 250 ha, spaced on a regular 1 × 1 km grid representing 5,000 ha. Past harvesting had removed the majority of old trees, so most samples were taken from stumps. In the following text, each sample means an individual tree.

### 2.3 Laboratory, statistical, and modeling analysis

Cross‐sectional samples were glued to wooden mounts and trimmed flat with a table saw or bandsaw. We then sanded cross sections with progressively finer grits of sandpaper until wood cells were clearly visible under magnification. Tree rings were visually cross‐dated (Stokes & Smiley, 1968) with a tree‐ring chronology from the Chuska Mountains (Guiterman, Swetnam, & Dean, 2016) and an unpublished local tree‐ring chronology that we developed. We measured tree rings to the nearest 0.001 mm with a Velmex measuring stage and Measure J2X software. COFECHA software (Grissino‐Mayer, 2001) was used to check the quality of cross‐dating. Many samples were difficult to cross‐date visually. Tree rings of these samples were measured as floating chronologies, and the COFECHA software was used to identify likely dates. All dating was confirmed visually, and all samples were checked by a second dendrochronologist. Fire‐scar locations within the annual growth rings were designated as: D (Dormant), EE (Early earlywood), ME (Middle earlywood), LE (Late earlywood), and L (Latewood) (Baisan & Swetnam, 1990). Following Baisan and Swetnam (1990), we interpreted intraring positions as being related to the timing in the growing or dormant seasons when the injury occurred; however, we acknowledge that these estimates are approximate. Some scar positions were labeled as “Undetermined” if the position was difficult to detect, especially in very narrow rings, or if the scarred area had been eroded by fire or weathering.

**Figure 1:**
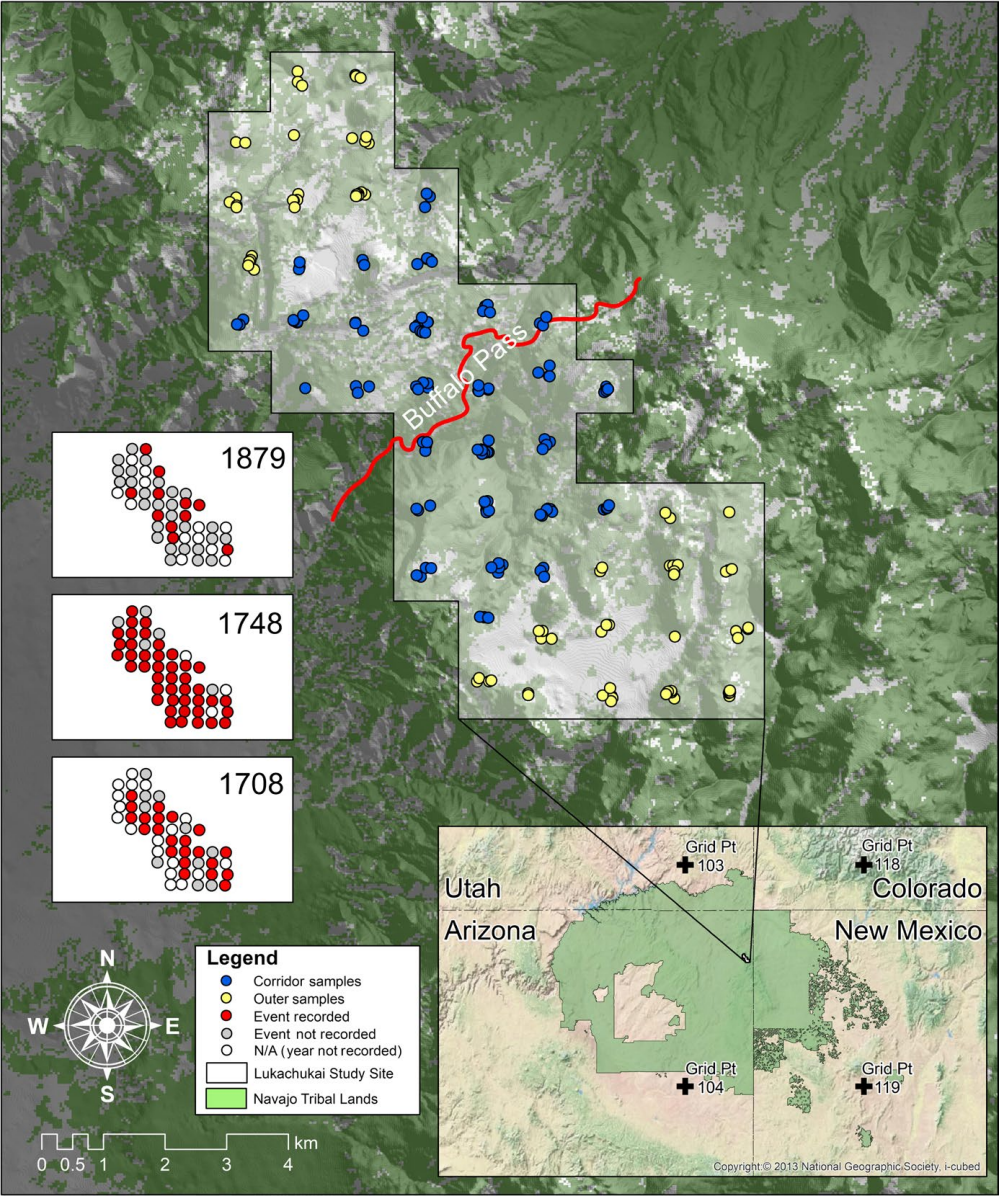
Study area in the Lukachukai Mountains, northern region of the Chuska Mountains. Fifty 5‐ha plots were sampled for fire‐scarred trees on a 1 × 1‐km grid. Colored dots show the position of individual fire‐scarred samples with blue shading representing Corridor and yellow shading representing Outer sites. Navajo Tribal Lands are shown within the “Four Corners” region, and the gridpoints 103, 104, 118, and 119 from the North American Drought Atlas (Cook & Krusic, 2004) are identified. The three subpanels (left) depict maps of fire occurrence for selected years (1708, 1748, and 1879) with red dots representing sites where at least one tree recorded a fire that year, grey dots represent sites where that year was present in the sample but no trees recorded a fire, and empty dots represent sites where that year was not present in the sample (i.e., the sample trees were all too young)

Fire history data were categorized in three groups: All (all 50 plots), Corridor (the 25 plots closest to the Buffalo Pass drainage), and Outer (the remaining 25 plots to the north and south, farther from the pass) (Figure 1). The pass corridor itself is relatively narrow but we divided the plots into two equal groups so that the fire histories could be compared on an equal‐area basis. We analyzed spatial and temporal fire patterns using FHAES software (Brewer, Velásquez, Sutherland, & Falk, 2016). We calculated fire regime statistics based on fire dates recorded by ≥2 fire scars recorded on different trees. Recording trees are those with an existing scar, making them relatively sensitive recorders of fire (Farris et al., 2013). In addition to considering all fire dates, we also applied proportional filters to assess fire years in which scarring affected ≥10% of the recording samples and ≥25% of the recording samples. The 10% and 25% scarred filters represent proportionately more widespread fires across the landscape (Farris et al., 2013). We applied the filters at the tree level (e.g., the ≥10%‐*scarred* filter required ≥21 trees of the 201 sample trees, as opposed to five of 50 plots) (Van Horne & Fulé, 2006) rather than compositing fire histories by plot (Farris et al., 2010; O′Connor et al., 2014). Either approach could be appropriate or both can be readily calculated from the online dataset at the International Multiproxy Paleofire Database (IMPD; https://www.ncdc.noaa.gov/data-access/paleoclimatology-data/datasets/fire-history). The method we used is relatively conservative, meaning that fire intervals tend to be longer for the 10% and 25% categories than if plot composites were used, because there is a higher minimum number of scarred samples to meet for each filter. We assessed the fire interval data with parametric statistics and also fit a Weibull distribution, which is often applied to fire interval distributions because they tend to be skewed (Grissino‐Mayer, 1999).

Fire and climate relationships were tested on fires that scarred a minimum of two trees and the all fires, 10%, and 25%‐scarred filter using Superposed Epoch Analysis (SEA) in the package “burnr” for R (Malevich, Guiterman, & Margolis, 2018). We used the SEA to determine the mean value of a climatic variable before, during, and after the years of fire events. The analysis period extended from 5 years prior to the fire year, the fire year, and 2 years after the fire. Statistical significance was determined with a 95% confidence interval using bootstrap methods with 1,000 simulations. The climate variables were a Palmer Drought Severity Index (PDSI) reconstruction averaged from the four nearest 2. 5° × 2.5° gridpoints (numbers 103, 104,118, and 119) from the North American Drought Atlas (Cook & Krusic, 2004). These four gridpoints are highly intercorrelated over the period 1644–1920, average *r* = 0.90. We also used the NINO3 reconstruction of the Southern Oscillation Index (Cook, 2000).

## 3 RESULTS

We collected 243 samples of *P. ponderosa* and five of *P. menziesii*; we cross‐dated and used 201 of the 248 collected samples (81%) in this study. The unused samples were undateable due to short, complacent, and/or decayed tree rings. The 201 fire‐scarred trees included two live trees, 35 snags, 45 logs, and 119 stumps. Results in the following sections are presented in three categories. The category “All” includes all 50 plots. The category “Corridor” comprises the 25 plots closest to the Buffalo Pass canyon, while the category “Outer” consists of the remaining 25 plots in the uplands farther from Buffalo Pass (Figure 1).

### 3.1 Fire regime changes

**Figure 2:**
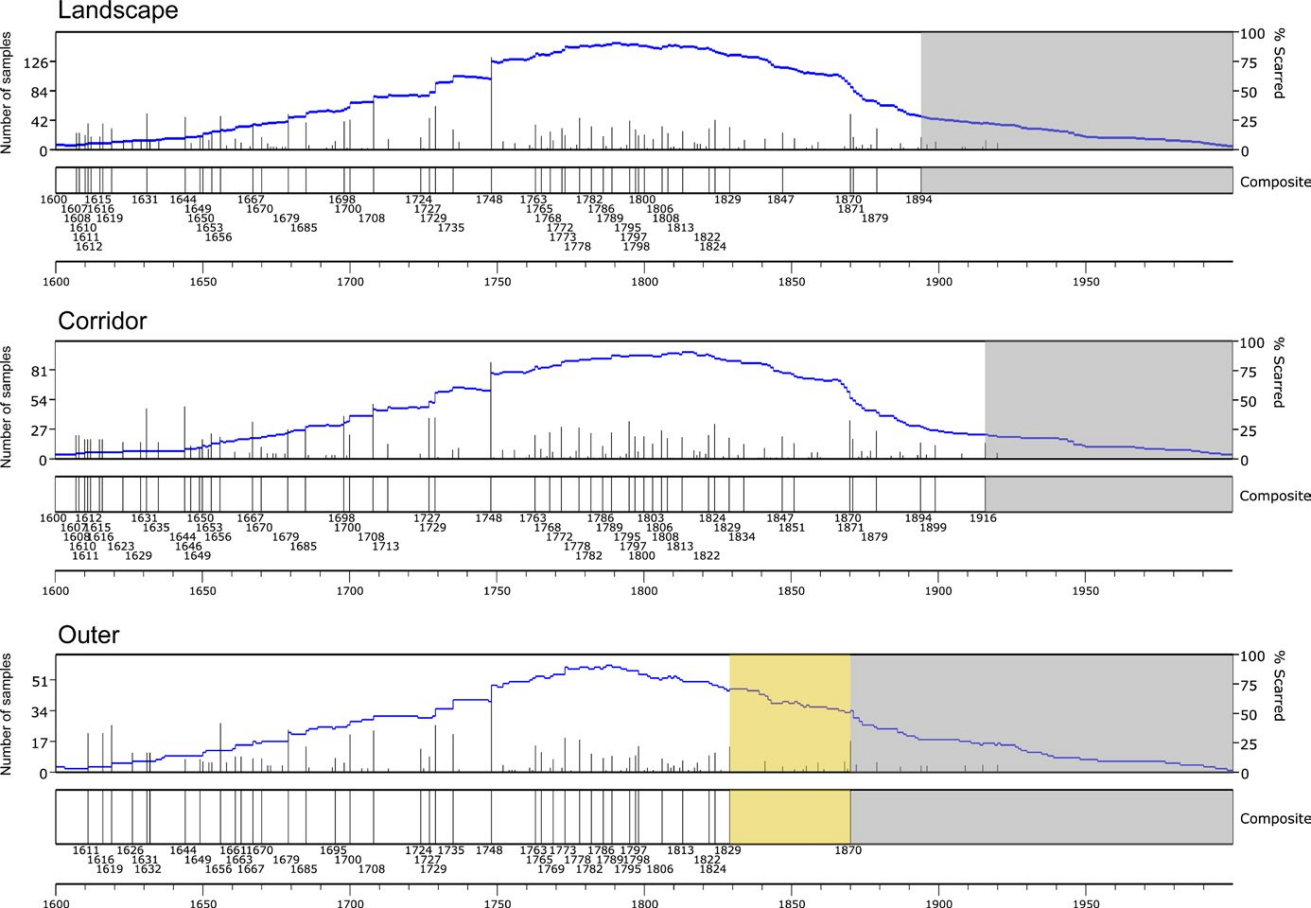
Fire events at Lukachukai study site in the categories All, Corridor, and Outer. Fires with dates shown on the *x*‐axis met the 10% fire‐scarred filter. The orange shading depicts the post‐1829 reduced fire interval in the outer region, and grey identifies the absence of fire events meeting the 10%‐scarred threshold

Fire occurrence within the full Lukachukai landscape (All 50 plots) was frequent until the late 19th century, after which there was a decline in fire events (Figure 2). At the scale of the full study area, therefore, our first hypothesis that fires would decline after ≈1830 was not confirmed. Nonetheless, a reduction in fire occurrence was apparent in the mid‐1800s compared to the preceding century (Figure 2), which we discuss below. The earliest fire dated from the 201 samples occurred in 1472; the final fire date was 1920. We assessed the fire interval distribution during the period 1644–1920, the earliest and latest fire dates representing a sample depth of 10% or more of the recording samples. The mean fire interval (MFI) for the full site ranged from 3.17 years for all fire events, approximately double (6.25 years) when using the 10%‐scarred filter, and 22.60 years using the 25%‐scarred filter. The minimum fire interval was 1 year for all‐scarred and 10%‐scarred filter and increased to 2 years when using the 25%‐scarred filter. A Weibull distribution model fit the data for all filters. The Weibull median probability interval (WMPI) was within <2.5 years of the MFI, a similarity which indicated that the fire interval distributions were not substantially skewed (Table 1).

**Table 1:** Fire return intervals (years)

Site/analysis period^a^	Category of analysis^b^	Number of intervals	Mean fire interval (MFI)	Minimum	Maximum	Weibull median fire interval
Landscape 1644–1920	All fires	87	3.17	1	17	2.66
	10%	40	6.25	1	23	5.06
	25%	10	22.60	2	46	19.94
Corridor 1644–1920	All fires	68	4.00	1	17	3.37
	10%	42	6.48	1	19	5.48
	25%	12	18.83	2	46	17.03
Outer 1644–1920	All fires	51	4.37	1	16	3.73
	10%	33	6.85	1	41	5.51
	25%	9	23.78	5	92	18.01

*Notes*

Sites are categorized as: Landscape, Corridor, and Outer sites, as described in text.

^a^Period of analysis is the time between the first and last fires recorded by a minimum of two trees scarred and ≥10% of recorder trees. ^b^Filters for 10% and 25% of trees scarred also include a minimum of two trees scarred and two trees recording.

**Figure 3:**
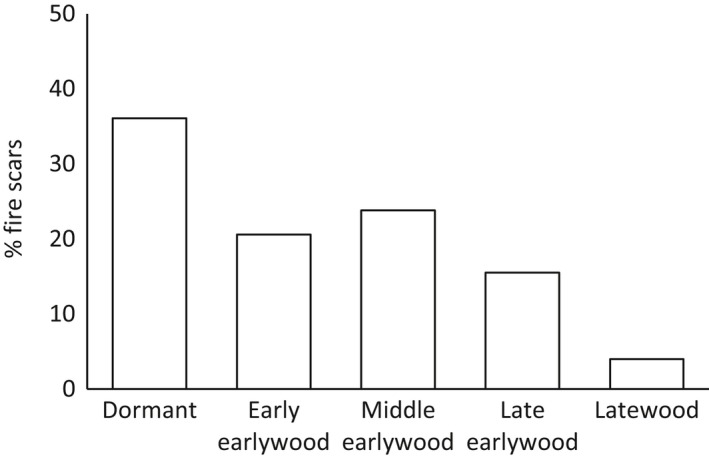
Seasonality of fire occurrence (10% fire‐scarred filter): 36% dormant, 20% early earlywood, 24% middle earlywood, 16% late earlywood, and 4% latewood

Seasonal position of the fire injury could be determined for 759 (66%) of the total of 1,153 fire scars (Figure 3). Most fires occurred in the dormant season (36%) or in the early (20%) to middle (24%) part of the growing season. Late earlywood accounted for 16% of scarring and latewood totaled 4% (Figure 3).

**Figure 4:**
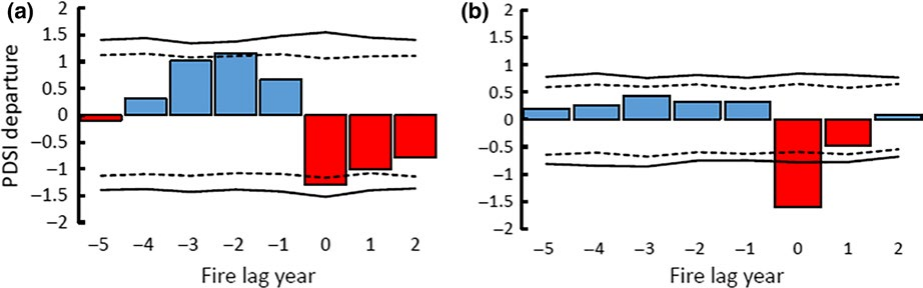
Superposed epoch analysis illustrating departures from the mean value of Palmer Drought Severity Index for years 1644–1916. (a) 25% or more fire‐scarred filter (minimum two fire‐scarred trees) applied with 0 representing fire year. Dotted lines represent 95% confidence interval derived from 1,000 Bootstrap simulations, and solid lines indicate those that exceed 99% confidence interval. (b) Same analysis with 10% fire‐scarred filter

### 3.2 Corridor versus outer regions

Differences in nineteenth‐century fire regimes were revealed by separating the landscape into the Buffalo Pass corridor versus the outer portion. Smaller fires occurred slightly more frequently in the corridor compared to the outer portion of the landscape, partially supporting our second hypothesis, as shown by the approximately 6%–10% shorter mean fire intervals for all fires and those scarring 10% of the samples (Table 1). In the 25%‐scarred category, the corridor had a 26% lower MFI when compared to the outer region.

More important, the landscape separation revealed a key spatial difference in terms of the initial fire regime interruption. Changes in fire regime were distinctly different between the regions: After 1829, the outer portion had only one fire that exceeded the 10% filter, in the year 1870. In contrast, the corridor had a pattern of frequent fires exceeding the 10% filter which continued until the last fire in 1916 (Figure 2). The difference in landscape patterns of burning after 1829 means that while the first research hypothesis was not confirmed across the full landscape, the outer region farther away from the Buffalo Pass corridor did experience a clear reduction in fire occurrence in this time period.

### 3.3 Fire–climate relationship

**Figure 5:**
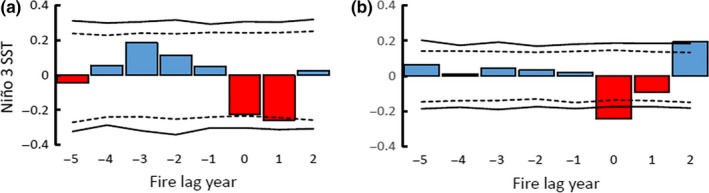
Superposed epoch analysis illustrating departures from the mean value of the Niño3 index for years (1644–1916). (a) 25% or more fire‐scarred filter (minimum two fire‐scarred trees) applied with 0 representing fire year. Dotted lines represent 95% confidence interval derived from 1,000 Bootstrap simulations, and solid lines indicate those that exceed 99% confidence interval. (b) Same analysis with 10% fire‐scarred filter

**Figure 6:**
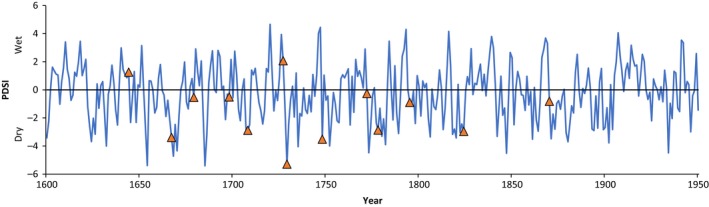
Palmer Drought Severity Index (PDSI) and fire event relationship for 25% fire‐scarred filter (triangles) in the Corridor site. The PDSI shown is the average of gridpoints 103, 104, 118, and 119 from the North American Drought Atlas (Cook & Krusic, 2004)

The SEA results showed significant interannual variability of drought as assessed by reconstructed PDSI before and during fire years (1644–1920). Fire years were generally preceded by wet conditions, switching to significantly dry conditions (negative PDSI) in the fire year and subsequent years. Fires were more likely to occur in La Niña years (negative values of NINO3, Figure 5). The 13 fire dates that met the 25%‐scarred filter across the entire study area were 1685, 1698, 1708, 1727, 1729, 1748, 1768, 1772, 1778, 1795, 1824, and 1870; ten of these thirteen fires occurred in years with notably below‐average PDSI values (Figure 6).

## 4 DISCUSSION

The historical fire regime of the Lukachukai Mountains bears many similarities to fire patterns reported in the Chuska Mountains and broadly across the Southwest. However, the spatially explicit landscape sampling method used in this study showed unique fine‐scale differences associated with topographic and land‐use features. The following sections discuss the three guiding hypotheses of the study.

### 4.1 H_1_: Fires declined in the 1830s due to pastoralism

From 1644, the earliest fire date with sufficient sample depth for statistical analysis, until 1920, the last fire date with ≥2 fire‐scarred samples, the landscape experienced frequent fires, with an MFI of 6.25 years for fire dates in which 10% or more of the recording samples were scarred and 22.6 years for the 25% filter. There was not a detectable reduction in fire frequency in the mid‐18th century at the scale of the entire landscape, although there were differences at finer scales, discussed in the following section.

Overall, the fire regime was broadly similar in terms of frequency and dates of fire regime shifts to those reported in nearby forests, albeit with the latest date of complete fire exclusion (1920) reported in the closest sites in the Four Corners region. Guiterman (2016) reported fire intervals in the 10%‐scarred category as ranging from 6.8 to 26.8 years at seven sites, including the site previously described by Savage and Swetnam (1990) in the Chuska Mountains. An additional site had a much longer MFI of 78.0 years (Guiterman, 2016). Several fire regime reconstructions have been carried out in mountains around the Chuska range. To the south in the Zuni Mountains (NM), Rother and Grissino‐Mayer (2014) reported MFI values (25%‐scarred) of 6.1–7.9 years in ponderosa forests with fires ceasing after 1880. To the northeast in the San Juan Mountains (CO), in relatively mesic mixed‐conifer forests, Grissino‐Mayer et al. (2004) found average fire return intervals of 21–30 years (final fire dates 1880 or 1890) and Fulé, Korb, and Wu (2009) reported MFI values (10%‐scarred) of 32.3 years (final fire in 1868). To the northwest, Heyerdahl, Brown, Kitchen, and Weber (2011) reported “plot‐composite low‐severity fire intervals” averaging 31 years in the Abajo Mountains (UT), with fire exclusion after about 1895. In summary, the Lukachukai Mountains fire regime was relatively similar in broad strokes to the regimes reconstructed from surrounding forests, despite specific details such as the early alteration of fire regime after 1830, the somewhat late fire interruption date of 1920, and the fire variation between the Buffalo Pass versus the outer uplands. In contrast to the notably different modern fire patterns on Hualapai Tribal lands (Stan et al., 2014) or San Carlos Apache Tribal lands (Pyne, 2014), the Lukachukai Mountains underwent a long fire‐free period after the early 20th century similar to that of southwestern public lands.

### 4.2 H_2_: Buffalo Pass corridor has a higher frequency of fire occurrence than surrounding outer uplands

The data supported the second hypothesis that the Buffalo Pass corridor had higher fire occurrence than the outer boundaries of the study site, although the difference had more to do with the temporal duration of the fire regime than the statistical attributes of fire frequency prior to 1830. Fires in the 10% fire‐scarred category on the outer boundary of the Lukachukai study site ceased after 1870 but in the Buffalo Pass corridor fires persisted until 1916 (the 1920 fire was recorded on at least two trees but did not reach the 10%‐filter threshold). Furthermore, the division of the landscape into corridor versus outer regions provided support for the pastoralism hypothesis, H_1_, because the fire regime in the outer region did decline notably after 1829. Only a single fire, in 1870, exceeded the 10%‐scarred threshold (Figure 2). In sum, the fire regimes of the corridor and outer regions were not distinguishable prior to 1829 but after that date the outer region underwent a fire reduction while the corridor continued to burn frequently. Burning in the corridor was sufficient to maintain the fire regime across the entire study area, causing us to reject the pastoralism hypothesis in the preceding section. However, in the outer region of the landscape, the reduction in surface fuel associated with pastoralism post‐1829 is a plausible factor in fire regime change (Savage & Swetnam, 1990).

The Buffalo Pass corridor and outer regions appear to provide a finer‐scale contrast in nineteenth‐century fire regimes than the differences observed at coarser scales by Guiterman (2016). Guiterman (2016) separated the Chuska and Defiance Plateau mountain ranges into areas that were likely used for livestock grazing as evidenced by the presence of water and settlements versus probably unused areas, showing that fires were reduced in used areas beginning around 1830 but not in unused areas until around 1880. At Buffalo Pass, we found both patterns, fire reduction and fire continuity, in close proximity. Furthermore, the entire Lukachukai landscape meets Guiterman′s criteria for a “used” site: There is water at least seasonally in the pass drainage as well as several springs and shallow lakes scattered throughout the uplands, while the pass provides access to well‐established settlements on either side.

Given that pastoralism is a plausible cause for the post‐1829 fire decline observed in the Lukachukai outer region, why does not the Buffalo Pass corridor exhibit this same trend? Fires continued in the corridor not only between 1830 and 1870 but also for several additional decades later. Our finding suggests that there are additional natural and human‐caused factors driving the prolonged fire events in the corridor. Differences in topographic position from the pass bottom to the ridge top and the “chimney effect” of wind drawn through a canyon have been documented as major influences in fire behavior in other southwestern forests (Holsinger, Parks, & Miller, 2016; Iniguez, Swetnam, & Yool, 2008). Possibly, there was more surface fuel in the Corridor plots due to more mesic conditions (shading, higher water table). The fact that people have moved through the Pass throughout the past, providing additional ignition sources, likely also contributed to prolonging fire in the Buffalo Pass corridor. In sum, even though grazing likely occurred in the Buffalo Pass corridor after 1830, as in the outer region, the fire‐favoring factors of topography and ignition may have maintained the fire regime for nearly a century after it was first interrupted circa 1830.

### 4.3 H_3_: Forest fires within the Lukachukai Mountains were synchronized with climatic variation

We found strong evidence that forest fires within the Lukachukai Mountains were synchronized by climate. Climatic oscillations that affected fires across the southwestern United States (Swetnam & Brown, 2011) were evident in the Lukachukai Mountains with the Palmer Drought Severity Index (PDSI) and El Niño‐Southern Oscillation (ENSO) being strong drivers of large‐scale fires. SEA results show that fires occurred primarily during significantly dry years and were preceded by above‐average wet years. This pattern is consistent with trends seen in other southwestern forests in which a buildup of surface fuels is antecedent to a switch to drier years (Swetnam & Baisan, 1996; Swetnam & Betancourt, 1998). The pattern of fire–climate synchrony at Buffalo Pass was highly similar to that reported by Guiterman (2016) in the nearby Chuska Mountains. Fire seasonality was broadly consistent with results from other southwestern sites, with most fires occurring in the dormant season and early to middle growing seasons, consistent with the premonsoonal dry period and onset of lightning storms. Patterns of human‐caused versus lightning‐ignited fires are difficult to distinguish in fire‐scar reconstructions (Kaye & Swetnam, 1999); we did not observe any distinct patterns that would permit identification of fire causes.

Within the period of study (1644–1920), our sites recorded approximately 50% of the largest fire years and only one of the smallest fire years (1649) captured in the southwestern United States from 1600 to 2000 (Swetnam & Brown, 2011). Comparing the largest fire years in the Southwest to large fires at our landscape (25% fire‐scarred filter), there are only eight fire years: 1698, 1708, 1727, 1768, 1772, 1778, 1795, and 1824 recorded in our site which do not coincide with other large fire years of the southwestern United States. The high synchrony of burning between the isolated Lukachukai/Chuska Mountains and other widely scattered southwestern sites is consistent with the argument by Swetnam and Betancourt (1990, 1998) that large‐scale synoptic climate forcing associated with ENSO entrains large‐scale patterns of fire (La Niña) and lack of fire (El Niño).

## 5 CONCLUSIONS

First, the fine‐scale differences between the corridor and the outer region on a 50 km^2^ landscape show that in addition to the top‐down driver (climate), there are bottom‐up drivers (fuel continuity, people, landscape configuration, etc.) affecting fire at local to landscape scales (Ireland, Stan, & Fulé, 2012). The results confirm differences in fire regime between Indigenous lands versus neighboring non‐Indigenous lands in the Southwest, a characteristic observed at other places worldwide (Mistry, Bibiana, & Berardi, 2016). Our findings underscore the needs for increasing knowledge on fire regimes and management practices using fire in Indigenous social‐ecological systems using paleoecological, historical, and social science research (Huffman, 2013; Trauernicht, Brook, Murphy, Williamson, & Bowman, 2015). Second, spatially explicit sampling of fire regime patterns over large landscapes reveals detailed patterns that may not be detected by coarser or finer‐scale approaches. Burning patterns interact with climate variation over space and time to produce a self‐limiting effect on fire characteristics (Parks, Parisien, Miller, Holsinger, & Baggett, 2018) that may be critical to avoid fuel buildup resulting in large crown fires such as the Asaayi Lake fire. Even the largest fire event—1748—recorded at 41 of the 50 gridpoints (also the largest historical fire year in the entire Southwest, Swetnam & Brown, 2011) left behind a legacy of fire‐surviving scarred trees at nearly every sampling point on the 1 × 1 km grid, showing that this large fire was not “stand‐replacing.” In contrast, the Asaayi Lake fire burned 59.5 km^2^ in the Chuska Mountains in 2014 with mostly lethal intensity, leaving large patches of complete tree mortality. Third, while the past fire regime of the Lukachukai Mountains was complex and affected by human activities at different spatial and temporal scales, since the early twentieth‐century fires have been completely excluded. This extended fire‐free period is unprecedented in the past several centuries in which fire history can be reconstructed from tree rings, and probably much longer back in ecological and evolutionary history (Anderson, Allen, Toney, Jass, & Bair, 2008). Since the last fire event to occur in the Lukachukai Mountains was approximately a century ago, forest managers should take account the wide departure from past fire patterns. It is likely that forest structural change and fuel accumulation due to fire exclusion, in combination with warming climate, present a hazardous situation favoring future severe burning. The loss of forest ecosystem services is particularly acute in Native communities due to the traditional cultural and spiritual value of the landscape, a high incidence of poverty which makes it difficult to substitute for local natural resources (Voggesser et al., 2013), and anticipated further losses due to warming climate (Azpeleta et al., 2014). Forest fuels treatments such as tree thinning and fire use, together with climate‐sensitive modeling of future forest change, are important to consider in guiding future management.

## CONFLICT OF INTEREST

None declared.

## AUTHOR CONTRIBUTIONS

PF, ASM, and YSK conceived the ideas. PF, LW, and AAT designed methodology. LW, PF, AAT, and ASM collected the data. LW, PF, and AAT analyzed the data. LW and PF led the writing of the manuscript. All authors contributed critically to the drafts and gave final approval for publication.

## DATA ACCESSIBILITY STATEMENT

Data are archived at the International Multiproxy Paleofire Database (IMPD; https://www.ncdc.noaa.gov/data-access/paleoclimatology-data/datasets/fire-history).
